# Survey of Wild and Domestic Mammals for Infection with *Leishmania infantum* following an Outbreak of Desert Zoonotic Visceral Leishmaniasis in Jiashi, People's Republic of China

**DOI:** 10.1371/journal.pone.0132493

**Published:** 2015-07-15

**Authors:** Chun-hua Gao, Jun-yun Wang, Song Zhang, Yue-tao Yang, Yong Wang

**Affiliations:** 1 National Institute of Parasitic Diseases, Chinese Center for Disease Control and Prevention, the Key Laboratory of Parasite and Vector Biology of the Chinese Ministry of Health, National Center for International Research on Tropical Diseases, WHO Collaborating Center for Malaria, Schistosomiasis and Filariasis, Shanghai, 200025, People’s Republic of China; 2 Center for Disease Control and Prevention of Xinjiang Uygur Autonomous Region, Urumqi, 830002, People's Republic of China; 3 Department of Immunology, School of Basic Medical Sciences, Central South University, Changsha, 410013, People's Republic of China; University of Minnesota, UNITED STATES

## Abstract

In 2008 and 2009, an outbreak of desert-subtype zoonotic visceral leishmaniasis occurred in Jiashi county, Xinjiang, China. So far, no animal reservoir has been identified for this type of visceral leishmaniasis. Therefore, we surveyed the most common mammals (wild and domestic) for *Leishmania* infections during the outbreak in 2008 and 2009 in order to identify the source of the visceral leishmaniasis in this region. Spleen, liver, bone marrow and blood samples collected from 86 wood mice (*Apodemus sylvaticus*), 61midday jirds (*Meriones meridianus*) and 27 Yarkand hares (*Lepus yarkandensis*) were tested for the presence of *Leishmania* by microscopy, culture and PCR. All of the animals were found to be negative for *Leishmania* infections; On the other hand, *Leishmania* DNA was detected in blood samples collected from livestock reared in the outbreak area: 30.36% (17/56) of sheep, 21.57% (11/51) of goats, 17.78% (8/45) of cattle, and 21.62 (8/37) of donkeys were positive for *Leishmania* DNA by PCR. The amplified kDNA sequences from the livestock samples matched *Leishmania* DNA sequences isolated from patients with visceral leishmaniasis in the study area. We suggest that these domestic mammals are a possible reservoir host for *Leishmania infantum* in the outbreak area.

## Introduction

Visceral leishmaniasis (VL) is a severe vector-borne disease caused by parasites of the *Leishmania donovani* complex (including *Leishmania donovani* and *L*. *infantum*) belonging to Trypanosomatidae family, Kinetoplastida order [1–3]. In China, about 530,000 VL cases occurred in 17 provincial-level administrative areas in 1951 [4]. The disease has been controlled in eastern China following the implementation of a national disease control program beginning in 1950s. However, the disease is still endemic in 61 counties in six provincial-level administrative areas in western China. In addition, the number of VL cases has increased in these endemic regions over the past years [5]. Furthermore, an outbreak of VL occurred in Jiashi county, Xinjiang Uygur Autonomous Region between 2008 and 2009. In this outbreak, the incidence rate (258 cases in 2008 and 207 cases in 2009) was over 20-fold higher compared to the average annual incidence (9.67 cases for the period 1996–2007). The majority of the cases (96.6%) were in children under the age of two [5,6].

Based on clinical symptoms and epidemiological characteristics, VL are divided into two main forms: zoonotic visceral leishmaniasis (ZVL), where the disease is transmitted from animals to humans, and anthroponotic visceral leishmaniasis (AVL), where transmissions occurs between humans [7,8]. Both types of VL are found in western China [5,6]. At present, AVL is only endemic in the plain oases of Kashi, Xinjiang, where the peridomestic sandfly *Phlebotomus longiductus* is the vector [5,6]. Most cases occur in young people, with a few cases also occurring in infants. ZVL can be further divided into the mountainous sub-type (MST-ZVL) and desert sub-type (DST-ZVL) [5,6]. The MST-ZVL is distributed in the mountainous and hilly regions of several provinces (Gansu, Sichuan, Shaanxi, and Shanxi). Most infected patients are children under the age of ten. Dogs have been identified as the major host for this sub-type. The infection rate of the canine population in the endemic regions can reach up to 69.6% [9,10]. The vector for MST-ZVL is the wild sandfly *Phlebotomus chinensis* [5,6]. DST-ZVL has been found in the desert areas of Xinjiang Uyghur Autonomous Region, western Inner Mongolia Autonomous Region, and northern Gansu Province. Most patients are infants and children under the age of three (more than 96% of cases occur in children < 2 years old) [5,6]. The wild sandfly *Phlebotomus wui* is the vector [5,6], but the source of the infection was not known. The outbreak of VL in Jiashi in 2008/2009was identified as the DST-ZVL on the basis of the etiologic agent (*L*. *infantum*) and epidemiological characteristics [6]. Cases of DST-ZVL usually occur between October and January of the following year with an incubation period of 1–6 months, whereas cases of AVL normally occur in two waves, the first one in April/ May and the second one in September/ October. As the sandfly season is between May and September, we speculated that an animal reservoir might exist[6].

Dogs are usually the principal reservoir for ZVL [7,8]. Yet, as only few dogs and other canids can be found in Jiashi, non-canine mammals may serve as the animal reservoir responsible for the outbreak of VL in this region. However, knowledge of the animal reservoir is crucial for planning any measures to control the disease. In order to identify animal reservoirs for DST-ZVL in Jiashi, we surveyed the most common wild and domestic mammals in the outbreak area for infection with *L*. *infantum*.

## Materials and Methods

### Ethics statement

This study was carried out in the township of Gholtoghrakh, Jiashi county, Xinjiang, P. R. China, which is not privately owned or protected. All sampling procedures and experimental manipulations were reviewed and approved by the Center for Disease Control and Prevention of Xinjiang Uygur Autonomous Region. Animal care and procedures were carried out in compliance with the Guidelines for the Care and Use of Laboratory Animals developed by the Institute of Parasitic Diseases, Chinese Center for Disease Control and Prevention. The study and its protocols were approved by the Ethics Committee of the Institute of Parasitic Diseases, Chinese Center for Disease Control and Prevention. Sodium pentobarbital anesthesia was administered for all surgery procedures in order to minimize suffering. This study did not involve human participants and no endangered or protected species was tested. Blood sampling from domestic animals was carried out with the verbal consent of the animals’ owners. The owners were informed about the study aims and procedures and agreed to participate in this research study.

### Study area

The study area (39.1°~40.0° N, 76.2°~78.0° E) is an arid desert with a typical arid-zone climate (an annual mean temperature of 12.7°C with maximum summer temperatures of 40°C and a mean annual rainfall of 45 mm) [6]. The vegetation ischaracteristic of the desert–forest landscape, consisting of *Populus diversifolia*, *Tamarix ramosissima*, and *Halimodendron halodendron*. Newly reclaimed desert oases also occur in the region [11].

### Wild mammal capture and sampling methods

The study area was originally uncultivated desert. Later humans populated the area and began to cultivate the land. There are no clear boundaries between villages and the desert. Data on the presence and distribution of wild mammals show that the predominant species in the village and adjacent desert are rodents. The most common desert animal is the Yarkand hare (*Lepus yarkandensis*), while canids such as wolves and foxes are rare in the study area. Thus, most of the animals captured and screened for *Leishmania* infection were rodents and hares.

Rodents were captured using the live traps with string door (23cm×8cm×10cm) baited with vegetables and peanut butter, which were placed within village and at the edges of village, and within cotton fields and scrublands surrounding the village in October and November of 2008 and 2009 (sandfly season is between May to September). Blood, bone marrow, liver and spleen tissue samples were taken from captured animals for screening for the presence of *Leishmania*. Liver and spleen samples were divided into three parts for microscopy, parasite culture and PCR. Smears from the spleen and liver samples were fixed with methanol. Samples for PCR were processed in a room exclusively designated for DNA handling. For culture, dissected tissue samples from liver, spleen and bone marrow were incubated in NNN medium in order to grow parasites.

Wild Yarkand hares were provided by villagers who captured the animals by driving them into nets in the desert near the village in October and November of 2008 and 2009. The animals were sacrificed by injection of sodium pentobarbital in the laboratory, and samples of blood, bone marrow, liver, and spleen samples were collected. The blood was mixed with ethylenediaminetetraacetic acid (EDTA) as an anticoagulant. Bone marrow, liver and spleen samples were processed in the same way as described for rodent samples.

### Domestic mammalian sampling

In the study area, the predominant domestic mammals are sheep, goats, cattle and donkeys, with very few dogs and cats. During October and November of 2009, blood samples (5 mL) were collected by venipuncture from the livestock by experienced veterinarians into tubes containing EDTA as an anticoagulant to preserve the whole blood for PCR. The tubes were immediately stored in a pre-chilled cooler and transferred to the laboratory on the same day. A later set of blood samples was collected from the animals with positive PCR results for retesting in April of 2010.

### Microscopic examination

Giemsa stained smears were examined using a 100x oil-immersion lens for the presence of *Leishmania* amastigotes. At least 2000 microscopic fields for each smear were examined by two experienced investigators to confirm negative results.

### Cultures

Bone marrow and pieces of liver and spleen were incubated in NNN medium for 6 months before being confirmed as negative. *In vitro* sub-inoculations were carried out monthly.

### PCR analysis of tissues samples

DNA extracts were prepared from spleen and liver for PCR screening following the methods described by Elnaiem D. A. *et al* [12]. Briefly, small portions of the preserved specimens (approximately 5 mm in diameter) of spleen and liver were cut into small pieces, lysed in buffer containing proteinase-K and subjected to standard phenol/

chloroform extraction and ethanol/salt precipitation procedures. Precipitated DNA was washed 3 times with 70% ethanol, air dried, dissolved in 100 ul of PCR water, and then stored at -20°C. DNA was also extracted from cultured *Leishmania* isolates including *L*. *infantum* MCAN/CN/90/SC isolated from a dog with VL in Sichuan Province (provided by Prof Hu Xiao-su from Sichuan University), *L*. *donovani* MHOM/CN/80/ 801 from a VL patient in an AVL-endemic area of Kashi, Xinjiang, and the *L*. *infantum* strains MHOM/CN/08/JIASHI-1; MHOM/CN/08/JIASHI-2; and MHOM/CN/08/JIASHI-5 isolated from DST-ZVL patients during an outbreak in 2008 in Jiashi, Kashi, Xinjiang [5]. The DNA was extracted from whole blood (1ml for a PCR reaction) and bone marrow samples using the E.Z.N.A. SQ Blood DNA Kit (Omega Bio-tech, Inc.).

*Leishmania* genus-specific oligonucleotide primers K13A (5’-dGTGGGGGAGGGGC GTTCT-3’) and K13B (5’- dATTTTACACCAACCCCCAGTT-3’) were used to test the samples for *Leishmania* DNA following the methods described by Wang Jun-yun [9]. For each reaction, 10 ng of genomic DNA from the five *Leishmania* isolates (MCAN/CN/90/SC, MHOM/CN/80/ 801, MHOM/CN/08/JIASHI-1, MHOM/CN/08/JIASHI-2 and MHOM/CN/08/JIASHI-5) was used as a positive control, and a negative control without *Leishmania* DNA was included. PCR products were analyzed using 2% agarose gel electrophoresis. Cloning and sequencing of PCR products were carried out following the methods described by Wang Jun-yun [9].

### Phylogenetic and molecular evolutionary analyses

DNA sequences were analyzed and aligned using ClustalX [13]. A phylogenetic tree was built using the sequences of amplified fragments obtained in this study and the 116-bp sequences from the different species of *Leishmania* downloaded from GenBank following previous described methods [9].

## Results

### *Leishmania* infection in captured wild animals in the study area

All of the 86 rodents captured in and around the village were identified as wood mice (*Apodemus sylvaticus*), while all 61 rodents captured in farmland and scrubland afar from the village were all identified as midday jirds (*Meriones meridianus*). Additionally, 27 Yarkand hares were captured in the desert near the village. All of the captured wild animals were negative for *Leishmania* infection (Table 1). No macroscopic lesions were found in any liver or spleen samples on postmortem examination.

**Table 1:** Examination of captured wild animals for the presence of *Leishmania* by microscopy, culture and PCR.

Species	Number	Samples	No. Positive sample (Total No. examined)		
			Microscopy	Culture	PCR assay
*Apodemus sylvaticus*	86	Spleen	0 (86)	0 (86)	n.d.*
		Liver	0 (86)	0 (86)	n.d.
		Bone marrow	n.d.	0 (86)	n.d.
*Meriones meridianus*	61	Spleen	0 (61)	0 (61)	0 (61)
		Liver	0 (61)	0 (61)	0 (61)
		Bone marrow	0 (61)	0 (61)	0 (61)
		Blood	n.d.	n.d.	0 (61)
*Lepus yarkandensis*	27	Spleen	0 (27)	0 (27)	0 (27)
		Liver	0 (27)	0 (27)	0 (27)
		Bone marrow	0 (27)	0 (27)	0 (27)
		Blood	n.d.	n.d.	0 (27)

*n.d., not detected

### *Leishmania* infection in domestic mammals

Blood samples from domestic animals including sheep, goats, cattle and donkeys were tested for *Leishmania* DNA by standard PCR. *Leishmania* DNA was detected in 30.4% of sheep samples, in 21.6% of goat samples, in 17.8% of cattle samples, and in 21.6% of donkey samples (Table 2).

**Table 2:** Detection of leishmanial DNA in blood samples of domestic mammals by PCR.

Animal species	Number	No. of Positive samples
Sheep	56	17
Goat	51	11
Cattle	45	8
Donkey	37	8

Five months later, new blood samples were collected from 29 animals with initial positive PCR results (12 sheep, 7 goats, 5 cows and 5 donkeys). The other 15 animals with positive PCR results had been sold or killed in the meantime. The blood samples of the retested animals gave again a positive PCR result.

### DNA sequence and phylogenetic analyses

Two PCR products amplified from two animals of each livestock species were selected at random for subcloning into the pGEM-T vector. In addition, PCR products amplified from the Chinese *Leishmania* isolates MCAN/CN/90/SC, MHOM/CN/80/801, MHOM/CN/08/JIASHI-1, MHOM/CN/08/JIASHI-2 and MHOM/CN/08/JIASHI-5 were also subcloned into the vector. Finally, five subclones from each PCR product were randomly selected for DNA sequencing. The sequences are listed in S1 Table. Alignment of sequences showed all PCR products were 116 bp long. The sequences of the amplified PCR products from animals from the four livestock species were identical to the three Chinese *Leishmania* strains from Jiashi, but were different to the Chinese *Leishmania* strains MCAN/CN/90/SC and MHOM/CN/80/801.

**Fig 1:**
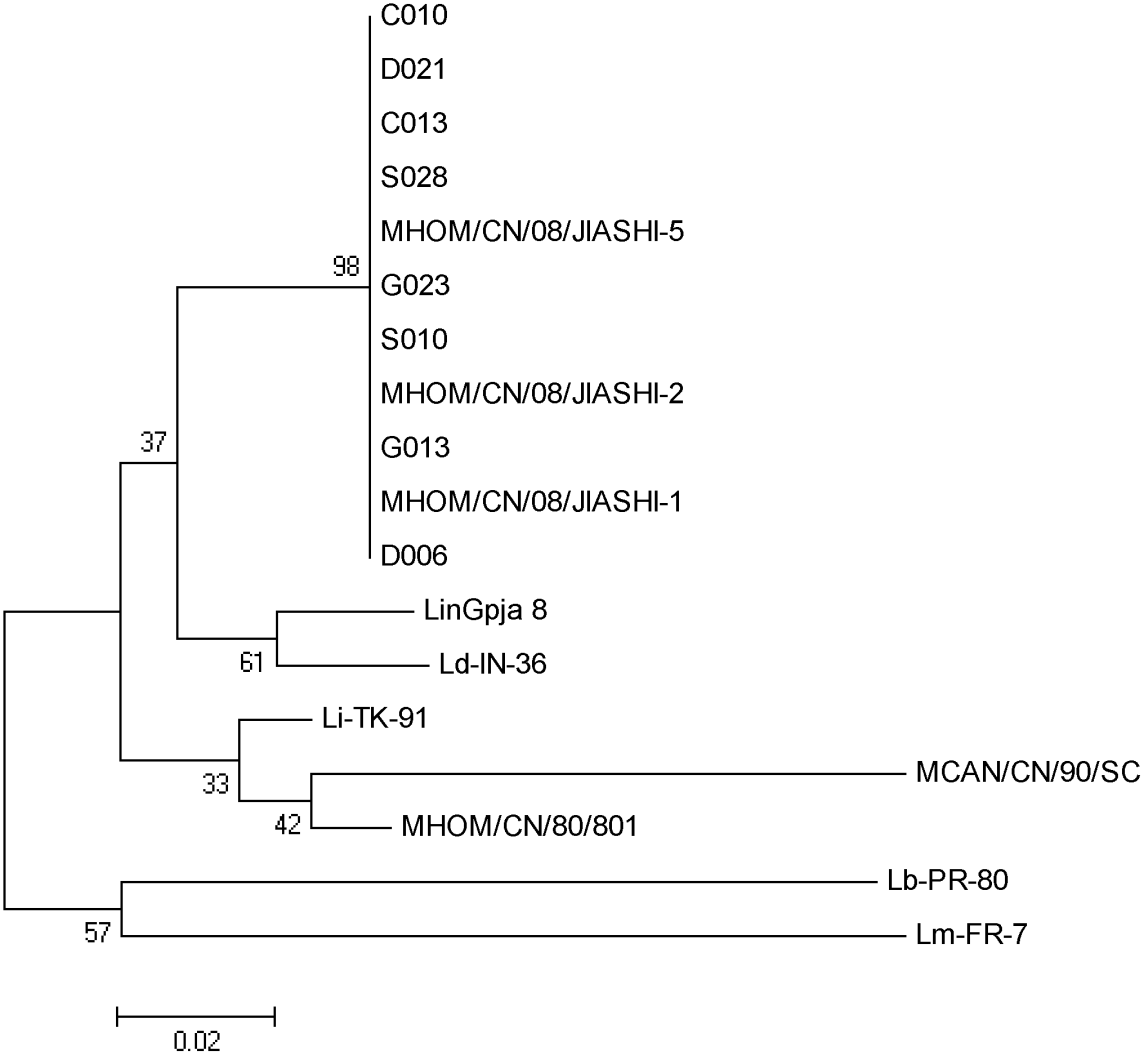
Dendrogram based on comparison of partial kDNA mini-circle sequences from *Leishmania* spp. The dendrogram was constructed using neighbor joining algorithm. Bootstrap analysis was done with 1000 replicates. Sequences included were amplified form blood samples obtained from cattle (C010 and C013), donkeys (D006 and D021), goats (G013 and G023), and sheep (S010 and S028), and from the Chinese *Leishmania* isolates MCAN/CN/90/SC, MHOM/CN/80/801, MHOM/CN/08/JIASHI-1, MHOM/CN/08/JIASHI-2 and MHOM/CN/08/JIASHI-5. The accession numbers of the *Leishmania* sequences extracted from GenBank are EU437406.1, EU370899.1, EU370886.1, EU370906.1 and EU370880.1 for the *L*. *infantum* strains LinGpja and Li-TK-91, the *L*. *donovani* strain Ld-IN-36, the *L*. *major* strain Lm-FR-7 and the *L*. *braziliensis* strain Lb-PR-80, respectively.

Based on the sequences obtained in this study and on other sequences from *Leishmania* species available in GenBank, a phylogenetic tree was constructed. As shown in Fig 1, sequences from the livestock animals and the three strains of *Leishmania* isolated from Jiashi, China, clustered together and were closely related to strains LinGpja 8 (*L*. *infantum*) and Ld-in-36 (*L*. *donovani*), but were less related to from other *Leishmania* strains including the Chinese isolates MCAN/CN/90/SC and MHOM/CN/80/801.

## Discussion

The source of desert sub-type of VL in China has not yet been identified. The existence of a zoonotic reservoir was suggested based on the following data: (1) most cases occurred between October and January [5,6], while the transmission season (adult sandfly activity season) is between May and September, making an outbreak of this form of VL by human-to-human transmission unlikely; (2) the causative agent of this sub-type of VL was identified as *L*. *infantum* [6]; and (3) desert sub-type VL is endemic in the northwestern desert of China, including Xinjiang, western Inner Mongolia and northern Gansu. These regions were uncultivated deserts and when humans populated these areas, they were affected by VL. Thus, the region was considered a natural reservoir for *Leishmania* parasites, with animals presumed to be the source for human infections [14].

To date, dogs have been considered as the main animal reservoir for ZVL, with other canids such as foxes and wolves playing a minor role [15–17]. Recently, some reports have indicated that wild rabbits (*Oryctolagus cuniculus*) could be reservoirs for *L*. *infantum* and may have been the source for an outbreak of VL in Madrid, Spain [18–22]. In addition, other animals, including cats [23–25], rodents, and the Egyptian mongoose (*Herpestes ichneumon*) [7] have been shown to be infected with *Leishmania*.

A VL reservoir host must fulfill several criteria, including abundance, distribution, longevity, and close association with both human and vector [26]. In the study area, the wild rodents wood mice and midday jirds are abundant and widely distributed. Yarkand hares are common in the desert (though they are seldom seen near villages), while wolves and foxes are rare. In this study, we surveyed wood mice, midday jirds and Yarkand hares and found no evidence of *Leishmania* infections in these animals based on microscopy, parasite culture or PCR. In a previous survey of an area endemic for desert sub-type VL, of 2482 captured wild animals (2183 wild rodents belonging to 9 genera, 114 foxes, 115 hedgehogs, 27 bats, 10 cats, 24 rabbits, and 9 shrews),none tested positive for *Leishmania* by parasitological examination [4]. The results of this study also showed no infection with *Leishmania* in any captured. This finding indicates that these wild animals are unlikely to be a reservoir hosts for *Leishmania* and thus not responsible for the outbreak of VL in the study area.

In the Jiashi County, domestic mammals including sheep, goats, cattle and donkeys are common, with sheep being the most abundant, while dogs and cats are rare. In this study, we found *Leishmania* DNA in all of the tested domestic species, especially in sheep (almost one third of sheep were tested positive for *Leishmania* DNA). Because DNA persists in the serum for only ~24 h [27], positive results of PCR become a good indicator of current or recent infection. Considering that transmission season in the study area is from May to September, our results thus indicated that these animals were infected with *Leishmania* for at least seven months. A recent study in Nepal proved that several months after the active transmission season, *Leishmania* DNA can still be detected in domestic animals such as goats, cows, and buffalos [28]. A comparison of 116-bp kDNA sequences indicated that the sequences obtained from livestock animals matched the sequences of 3 *Leishmania* isolates obtained from local VL patients during the outbreak but were different from other *Leishmania* isolate sequences obtained from somewhere else in China. These results suggest that the *Leishmania* strain carried by domestic animals is the same strain infecting humans in the study area.

Because over 90% of the VL patients in DST-ZVL areas were children under the age of 2 who usually do not move far away from their home., the source of infection must have been present in or around the village during the outbreak. This paper is the first report of the presence of *L*. *infantum* DNA in domestic mammals (sheep, goats, cattle and donkey) in an area with endemic desert-type VL. We suggest that these domestic mammals are likely the reservoir hosts for *L*. *infantum* in the endemic area. Isolation of the *Leishmania* parasites from domestic animals and testing their infectivity for the sandfly vector is required to elucidate the role of livestock as host reservoir in VL transmission to human in the study area.

## Supporting Information

S1 Table Sequences amplified using Leishmania genus-specific oligonucleotide primers K13A and K13B. (DOC)
