# Supplementary material for: Survey of Wild and Domestic Mammals for Infection with Leishmania infantum following an Outbreak of Desert Zoonotic Visceral Leishmaniasis in Jiashi, People's Republic of China
Source: PLoS One. 2015 Jul 15;10(7):e0132493. doi: 10.1371/journal.pone.0132493 (PMC4503304; doi:10.1371/journal.pone.0132493)
Supplement: S1 Table — (DOC) [file pone.0132493.s001.doc]

Supporting Information

**S1 Table. Sequences amplified using *Leishmania* genus-specific oligonucleotide primers K13A and K13B**

| Sequence ID | Sequence |
| --- | --- |
| Lb-PR-80 | GGGTAGGGGCGTTCTGCGAAATTCGGAAAAATGAGTGCAGAAACCCCGTTCATAATTTGGCGGGAAACCCCGAAATTCGGCTCCGGGCGGGGAAACTGGGGGTTGGTGTAAAAT |
| Lm-FR-7 | GGGTAGGGGCGTTCTGCGAAAACCGAAAAATGGGTGCAGAAATCCCGTTCATTTTTGGTCGGAAAACGCCATTTTTGGGCTCGGAGGCGGGAAACTGGGGGTTGGTGTAAAAT |
| LinGpja_8 | GGGTAGGGGCGTTCTGCGAAAATCGAAAAATGGGTGCAGAAATCCCGTTCAAAAATTGGCCAAAAATGCCAAAAATGGGCTCCGAGGCGGGAAACTGGGGGTTGGTGTAAAAT |
| Li-TK-91 | GGGTAGGGGCGTTCTGCGAAATCGGAAAAATGGGTGCAGAAATCCCGTTCAAAAATCGGCCAAAAATGCCAAAAATCGGCTCCGGGGCGGGAAACTGGGGGTTGGTGTAAAAT |
| Ld-IN-36 | GGGTAGGGGCGTTCTGCGAAAATGGAAAAATGGGTGCAGAAATCCCGTTCAAAAAATAGCCAAAAATGCCAAAAATCGGCTCCGAGGCGGGAAACTGGGGGTTGGTGTAAAAT |
| C010 | GTGGGGGAGGGGCGTTCTGCGAAAATGGAAAAATGGGTGCAGAAATCCCGTTCAAAAATCGGCGGAAAATGCCAAAAATCGGCTCCGAGGCGGGAAACTGGGGGTTGGTGTAAAAT |
| C013 | GTGGGGGAGGGGCGTTCTGCGAAAATGGAAAAATGGGTGCAGAAATCCCGTTCAAAAATCGGCGGAAAATGCCAAAAATCGGCTCCGAGGCGGGAAACTGGGGGTTGGTGTAAAAT |
| D006 | GTGGGGGAGGGGCGTTCTGCGAAAATGGAAAAATGGGTGCAGAAATCCCGTTCAAAAATCGGCGGAAAATGCCAAAAATCGGCTCCGAGGCGGGAAACTGGGGGTTGGTGTAAAAT |
| D021 | GTGGGGGAGGGGCGTTCTGCGAAAATGGAAAAATGGGTGCAGAAATCCCGTTCAAAAATCGGCGGAAAATGCCAAAAATCGGCTCCGAGGCGGGAAACTGGGGGTTGGTGTAAAAT |
| G013 | GTGGGGGAGGGGCGTTCTGCGAAAATGGAAAAATGGGTGCAGAAATCCCGTTCAAAAATCGGCGGAAAATGCCAAAAATCGGCTCCGAGGCGGGAAACTGGGGGTTGGTGTAAAAT |
| G023 | GTGGGGGAGGGGCGTTCTGCGAAAATGGAAAAATGGGTGCAGAAATCCCGTTCAAAAATCGGCGGAAAATGCCAAAAATCGGCTCCGAGGCGGGAAACTGGGGGTTGGTGTAAAAT |
| S010 | GTGGGGGAGGGGCGTTCTGCGAAAATGGAAAAATGGGTGCAGAAATCCCGTTCAAAAATCGGCGGAAAATGCCAAAAATCGGCTCCGAGGCGGGAAACTGGGGGTTGGTGTAAAAT |
| S028 | GTGGGGGAGGGGCGTTCTGCGAAAATGGAAAAATGGGTGCAGAAATCCCGTTCAAAAATCGGCGGAAAATGCCAAAAATCGGCTCCGAGGCGGGAAACTGGGGGTTGGTGTAAAAT |
| MHOM/CN/08/JIASHI-1 | GTGGGGGAGGGGCGTTCTGCGAAAATGGAAAAATGGGTGCAGAAATCCCGTTCAAAAATCGGCGGAAAATGCCAAAAATCGGCTCCGAGGCGGGAAACTGGGGGTTGGTGTAAAAT |
| MHOM/CN/08/JIASHI-2 | GTGGGGGAGGGGCGTTCTGCGAAAATGGAAAAATGGGTGCAGAAATCCCGTTCAAAAATCGGCGGAAAATGCCAAAAATCGGCTCCGAGGCGGGAAACTGGGGGTTGGTGTAAAAT |
| MHOM/CN/08/JIASHI-5 | GTGGGGGAGGGGCGTTCTGCGAAAATGGAAAAATGGGTGCAGAAATCCCGTTCAAAAATCGGCGGAAAATGCCAAAAATCGGCTCCGAGGCGGGAAACTGGGGGTTGGTGTAAAAT |
| MCAN/CN/90/SC | GTGGGGGAGGGGCGTTCTGCGAAATCGGAAAAATGGGTGCAGAAATCCCGTTCAAAAAATGCCCAAAAATGCCAATTTTGGCCTCCGGGGCGGAAAACTGGGGGTTGGTGTAAAAT |
| MHOM/CN/80/801 | GTGGGGGAGGGGCGTTCTGCAAAATCGGAAAAATGGGTGCAGAAATCCCGTTCAAAAATTGGCCAAAAATGCCAAAAATCGGCTCCGGGGCGGGAAACTGGGGGTTGGTGTAAAAT |
